# DGW: an exploratory data analysis tool for clustering and visualisation of epigenomic marks

**DOI:** 10.1186/s12859-016-1306-0

**Published:** 2016-12-13

**Authors:** Saulius Lukauskas, Roberto Visintainer, Guido Sanguinetti, Gabriele B. Schweikert

**Affiliations:** 1 Department of Chemical Engineering, Imperial College London, London, SW7 2AZ UK; 2 School of Informatics, University of Edinburgh, 10 Crichton St, Edinburgh, EH8 9AB Scotland; 3 Fondazione Bruno Kessler, Via Sommarive 18, Povo, I-38123 TN Italy

**Keywords:** Clustering, ChIP-seq, Epigenetics, Dynamic Time Warping

## Abstract

**Background:**

Functional genomic and epigenomic research relies fundamentally on sequencing based methods like ChIP-seq for the detection of DNA-protein interactions. These techniques return large, high dimensional data sets with visually complex structures, such as multi-modal peaks extended over large genomic regions. Current tools for visualisation and data exploration represent and leverage these complex features only to a limited extent.

**Results:**

We present DGW, an open source software package for simultaneous alignment and clustering of multiple epigenomic marks. DGW uses Dynamic Time Warping to adaptively rescale and align genomic distances which allows to group regions of interest with similar shapes, thereby capturing the structure of epigenomic marks. We demonstrate the effectiveness of the approach in a simulation study and on a real epigenomic data set from the ENCODE project.

**Conclusions:**

Our results show that DGW automatically recognises and aligns important genomic features such as transcription start sites and splicing sites from histone marks. DGW is available as an open source Python package.

## Background

**Fig. 1:**
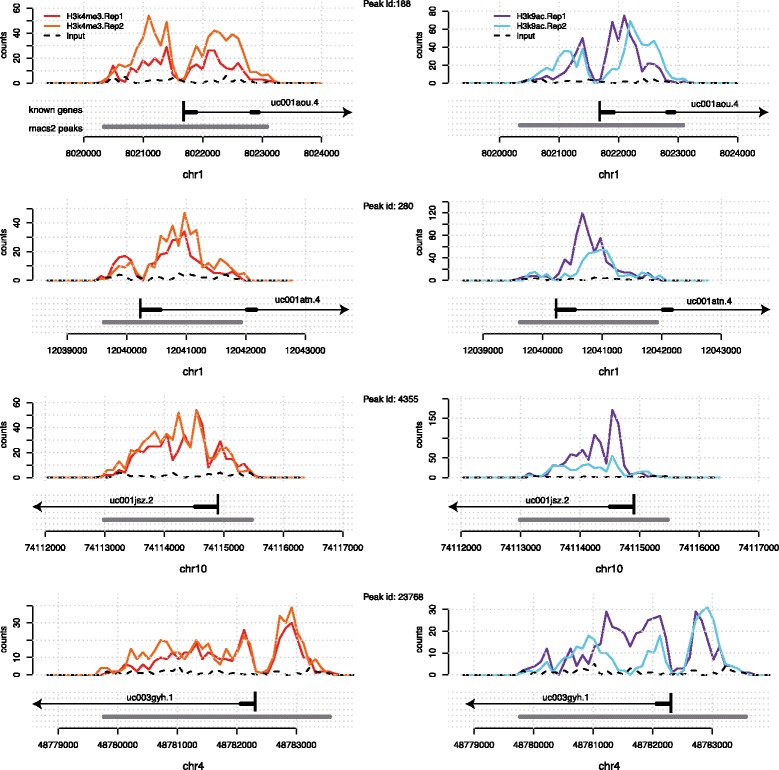
The epigenomic marks H3K4me3 (*left*) and H3K9ac (*right*) accumulate around transcription start sites often showing a bimodal peak with a valley over the TSS. Shown are two biological replicates for each mark and the input signal. Y axis corresponds to read counts. Annotated genes and the enriched regions called by MACS2 are shown in *grey* below each profile

Sequencing based technologies such as ChIP-Seq and DNAse-Seq [e.g. reviewed in [1]] have revolutionized our understanding of chromatin structure and function, yielding deep insights in the importance of epigenomic marks in the basic processes of life. The emergent picture is that gene expression is controlled by a complex interplay of protein binding and epigenomic modifications. While histone marks (and other epigenomic marks) can be measured in a high throughput way, exploratory data analysis techniques for these data types are still being developed. Epigenomic marks exhibit characteristics that distinguish them fundamentally from e.g. mRNA gene expression measurements: they are spatially extended across regions as wide as several kilobases within which they often present interesting local structures, such as the presence of multiple peaks and troughs [2], and intriguing asymmetries [3] (see Fig. 1). The shape of epigenomic marks across replicate data sets appears to be highly conserved, and has recently been exploited for statistical testing [4]. While the biological reasons for such conservation are not entirely clear, recent studies have suggested that both architectural and regulatory aspects may be at play. Bieberstein and colleagues showed intriguing patterns of accumulation of the histone marks H3K4me3 and H3K9ac at splice sites [5], hinting at an architectural origin of the shape of the marks. More recently, Benveniste et al showed that histone marks can be very well predicted genome-wide by the binding patterns of transcription factors (TFs) [6]. The shape of the peak may therefore be a readout of additional chromatin-related events and genomic regions which are similarly marked may therefore hint at common regulatory or architectural features. Excellent visualisation tools (e.g. UCSC genome browser) enable researchers to appreciate such features for individual enrichment peaks. However, while automatically grouping such marks based on shape similarity may be a valuable tool for hypothesis generation, it has remained a non-trivial task.

Current approaches to clustering regions based on chromatin signatures can be broadly split into two camps: global approaches, such as the celebrated HMM-based reconstruction of the “colours of the chromatin” [7], try to find a segmentation of large genomic regions based on histone signatures. These approaches usually rely on the presence vs absence characterization of histone marks at genomic loci, such that the clustering is primarily based on combinatorial patterns of multiple histone marks, as opposed to spatial patterns emerging within individual peaks. Another interesting segmentation approach was recently introduced by Knijnenburg and colleagues [8]. Here, signal enrichment is considered across a wide range of scales spanning several orders of magnitude. While this constitutes a significant improvement compared to earlier approaches, signal patterns within segments are again not taken into account. On the other hand, local approaches attempt to cluster short genomic regions at particular loci based on the quantitative binding or modification pattern measured at the loci (e.g. via ChIP-Seq). Examples of these approaches include the ENCODE Cluster Aggregation Tool (CAGT) [3], or the clustering of genes based on PolII binding profiles performed in [9]. Local approaches have to address two challenging problems: aligning the peaks to a reference, and standardising the peaks so that they can be represented as vectors of equal dimensions. To align regions, both, the method by Taslim and colleagues as well as the CAGT tool, rely on anchor points (e.g. transcription start sites (TSS) [9] or transcription factor binding sites from auxiliary ChIP-Seq experiments [3]). The regions are then standardised either by rescaling to a fixed gene length [9] or by applying windows of fixed length either side of the anchor points [3] irrespective of the true extent of the local enrichment. These strategies may be plausible for certain applications. However, the shape and extent of histone marks for instance, appear to be determined by many factors [5], such that a uniform rescaling may be inappropriate. In particular, if one made the assumption that epigenomic marks are directly or indirectly influenced by the underlying DNA sequence, it becomes clear that more flexibility in the comparison and alignment of these marks is needed: for example, ortholog genes may share similar sequence features but their sequence length may vary. Sequence comparisons therefore in general do not require the considered sequences to be of equal length, they allow for insertions, deletions, shifts. Similar local variations should therefore be allowed when comparing epigenomic marks.

In this work, we address the problem of aligning and clustering epigenomic data in a completely unsupervised way: as input data we use ChIP-Seq enrichment measurements within peak regions identified by a peak finder such as MACS [10]. The alignment and the standardisation problems are solved simultaneously without the use of additional information, such as transcription start sites or gene annotation. We introduce a local rescaling which allows to match epigenomic marks based on maximum similarity between shapes. Our method, Dynamic Genome Warping (DGW), is based on the classical Dynamic Time Warping algorithm [11,12], which enabled computer scientists to construct robust speech recognisers undeterred by the variability in pitch and speed of enunciation. In DGW we have implemented multidimensional alignment and clustering, such that multiple epigenomic tracks can be analysed simultaneously. This feature can also be used to control for local sequencing bias as DNA inputs or IGG controls can easily be added to the analysis. We first test DGW in a simulation study. Subsequently, we demonstrate that DGW can align genomic landmarks such as TSSs and first splicing sites (FSSs) on real epigenomic data from the ENCODE project [13], thus effectively and automatically solving both the alignment and the standardization problems. DGW is freely available as a stand-alone, platform-independent and fully documented Python package.

## Methods

We will first motivate and illustrate our method on a particular data set of histone modifications from the ENCODE project [13], measuring tri-methylation of histone 3 at lysine 4 (H3K4me3) and acetylation of histone 3 at lysine 9 (H3K9ac) in human leukaemia cell line K562. The reason for choosing these two specific marks is that they are known to be characteristically enriched in the flanking regions of TSSs [2] and they were recently shown to accumulate at FSSs [5], hence providing direct evidence of the biological relevance of both the alignment and standardisation problems.

Aligned fragments (BAM files) of both epigenomic marks were processed with the MACS2 peak caller [10] to identify regions which showed enrichment relative to a input control sample; we then merged the two sets by considering every region called for either mark. We stress that the method is independent of the specific marks chosen, or the choice of peak caller, and is readily extendable to other types of genomic and epigenomic data.

Enriched regions normally have very different lengths, nevertheless visual inspection of peaks can reveal similarities between the shape of the peaks. These similarities are often visualised through a global averaging (aggregation) of the marks as in [2], nevertheless there are strong arguments that global averaging may also mask more subtle patterns. A useful motivating example is given in Fig. 1. This shows four regions which are enriched in the H3K4me3 as well as H3K9ac marks. They all overlap with genes and exhibit broadly similar shapes: a bimodal peak with a trough over the TSS. However, the total lengths of the enriched regions vary, and so does the extent of the two individual sub-peaks, which could be governed by the underlying gene structure. Therefore, the position of the TSS relative to the start of the enriched regions varies.

### Dynamic genome warping

To automatically quantify the similarities between peaks such as the ones shown in Fig. 1 we use the classic Dynamic Time Warping (DTW) algorithm [11,14]. A modern review of the basic concepts of Dynamic Time Warping can be found e.g. in [12]. It was originally introduced in the speech recognition community to robustly recognize speech independently of speech speed. There, the problem was to match waveforms of similar shape but potentially different duration. Likewise, our aim is to be able to associate peaks which exhibit similar local structure (shape) regardless of their spatial extension.

Specifically, let **a**=(*a*_1_,…,*a*_*N*_) and **b**=(*b*_1_,…,*b*_*M*_) be two sequences with values $a_{i},b_{i} \in \mathcal {S}$, where $\mathcal {S}$ is a metric space equipped with local distance $d\colon \mathcal {S}\times \mathcal {S}\rightarrow \mathbb {R}$ (e.g. squared Euclidean distance or Cosine distance). DTW uses dynamic programming to construct a *warping path* $\mathbf {p}=\left ({p_{1}^{0}},{p_{1}^{1}}\right),\ldots,\left ({p_{i}^{0}},{p_{i}^{1}}\right),\ldots,\left ({p_{K}^{0}},{p_{K}^{1}}\right)$, i.e. two sets of indices identifying the elements of the two sequences which are mapped to each other in order to minimise the sum of the local distances. In formulae,

1 $$ \mathbf{p}=\text{argmin}\sum\limits_{i=1}^{K}d\left(a_{{p_{i}^{0}}},b_{{p_{i}^{1}}}\right)  $$

subject to the following constraints

- ${p_{1}^{0}}={p_{1}^{1}}=1$, the first points of both sequences are mapped to each other;
- ${p_{K}^{0}}=N$, ${p_{K}^{1}}=M$, the end points of both sequences are mapped to each other;
- $0\le p_{i+1}^{j}-{p_{i}^{j}}\le 1$ for all *i*=1,…,*K* and *j*=0,1, each index set is non-decreasing with maximum step one. This ensures that every point in each sequence gets mapped to at least one point on the other sequence.

**Fig. 2:**
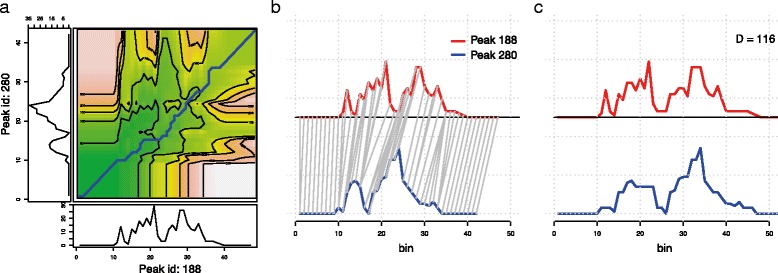
DGW alignment of two H3K4me3 profiles. **a** Shown is the distance density matrix for two peaks (Peak 188 on the x-axis and Peak 280 on the y axis). Colour coding corresponds to local Euclidean distances from small (*green*) to large (*red*). Optimal path is shown in blue. **b** Mapping between the two profiles. **c** Dynamically aligned profiles and total distance *D* between the two peaks

Algorithmically, DTW is very similar to the classical alignment algorithms such as Needleman-Wunsch and Smith-Waterman: it assumes an optimal alignment between subsequences, iterates by selecting the optimal next move and recovers the optimal global alignment by backtracking. As such, it entails constructing a matrix of size *M*×*N*, which determines the computational complexity of the algorithm: Computing pairwise DTW distances between all peaks is therefore the computationally most expensive step, as it involves computing $O\left (N_{peaks}^{2}\right)$ DTW distances, each of which is *O*(*M*×*N*). In Fig. 2 we show how the first two peaks in Fig. 1 are aligned onto each other using DTW. Notice that the pure DTW algorithm allows arbitrarily long stretches to be compressed to a single point. This behaviour may be undesirable, and simple modifications are implemented such as an upper limit on the length of compressed regions (Sakoe-Chiba band [11]), or an exponential penalty on compressing/stretching. By applying the Sakoe-Chiba Band constraint we can also reduce the run-time to *O*(*k*×*m* *a* *x*(*N*,*M*)), where k is the width of the band, that can be chosen to be small. Novel strategies to reduce the computational load are however emerging [15], and it would be interesting to integrate such ideas in the epigenomic context.

DGW readily extends to multi-dimensional data if more than one epigenomic track is analysed: In this case **a** and **b** become sequences of vectors, e.g. (**a**_1_,…,**a**_*N*_), that each contain the coverage of each mark at time point *i*. In this way, the different epigenomic marks are given equal weight, however other weighting schemes can easily be implemented.

In addition to the optimal path between two sequences we also report their total distance under the optimal warping which will subsequently be used for the clustering of peaks. Note, when using squared Euclidean distance as local distance measure, both, differences in peak shapes as well as in enrichment levels contribute to the overall DTW distance. If this is not desired the peaks can optionally be normalized by the respective peak heights, and the Cosine distance can be used as local distance. To account for potential strand specificity of epigenomic marks we compute two distances for every pairwise peak comparison: one with the two sequences unchanged, and one with one of them reversed. The smaller distance between the two is then returned as the true distance between the patterns.

### Clustering

After aligning all pairwise distances between peaks, we next aim to cluster them into groups which share similar shapes. Implementing k-means clustering within a DTW framework, however, would require the ability to define an average of all potentially possible warped profiles, which is not an easy task. Instead we take advantage of the pre-computed pairwise distances between peaks and perform agglomerative hierarchical clustering, using complete linkage to avoid chaining [16]. The resulting dendrogram contains *N*_*peaks*_−1 nodes, each of which represents a possible clustering of the data. As in any hierarchical clustering method, the number of clusters can be adaptively chosen by the user. This is both a strength and a weakness of the methodology. Principled methods for choosing a cutoff exist [17] and implementing them in the context of DGW will be a future direction of improvement. DGW computes a prototype for each node, i.e. a sequence representative of all sequences attached to the node (leaves of the tree which has the chosen node as a root). Prototype computation is a non-trivial problem in DTW; here we use the scaled prioritised shape averaging algorithm of [18].

### Pre-processing pipeline and implementation

Here we briefly describe the DGW software package; a more thorough description, including installation instructions and examples, is given in the vignette at the DGW home-page [19]. DGW consists of two modules: a worker module, which performs the computationally intensive tasks, and an explorer module, which allows visual exploration of the results. DGW-worker takes as input a set of genomic regions (a bed file e.g. returned by a peak finder) and a set of data files (bam files) for different epigenomic marks. Single-end reads are extended to the estimated fragment lengths. To alleviate the computational burden and to reduce spatial noise, coverage within peak regions are binned into non-overlapping windows spanning 50 bp. This is an adjustable parameter which should reflect the scale at which local changes are expected in the data. For each peak, we thus construct a sequence **a**=(*a*_1_,…,*a*_*N*_), which contains as values *a*_*i*_ the coverage within each bin *i*. At this point, we do not normalize with the input sample but use simple read counts. A practical reason for this is that most input samples still have a relatively low coverage. As there is no enrichment for binding sites, input samples cover the whole genome. Input library sizes therefore need to be significantly larger than their IP sample counterparts, which in practice is rarely the case. A simple correction, which uses enrichment over input is in most applications counterproductive as it adds additional noise to the signal. However, our method allows to add input samples for multidimensional clustering offering a convenient way to incorporate the additional information which is conveyed in a sufficiently sequenced input sample if it is available. The DGW-worker then computes the warping distances, the hierarchical clustering dendrogram, and the prototype sequences associated with each node; this is computationally intensive and the tasks will be automatically distributed across multiple cores if available. A typical run of DGW worker on the ChIP-seq data set takes 420 mins of CPU time distributed across six cores, for a total execution time of just over one hour.

Once these computations are completed, the lightweight explorer module can be launched. This opens a window displaying a heat-map of the peaks and the clustering dendrogram. The dendrogram can be cut at any desired level. The information about which peaks are clustered is returned as a series of BED files (one per cluster) to enable subsequent analyses. Individual clusters can be further analysed and additional functionalities are provided at this level, e.g. histograms of the positions of specific regions of interest pre and post warping (Fig. 6) and warpings of individual peaks onto prototypes can be obtained.

## Results and discussion

### Simulation study

**Fig. 3:**
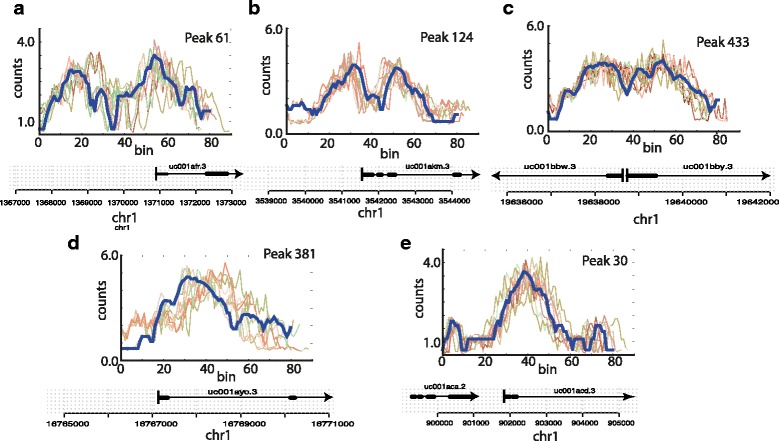
Generation of simulated data sets: Shown in *blue* are five *seed regions*, i.e. original ENCODE H3K4me3 read counts at the start of five known genes. For each of the seed regions we show 10 simulated modifications which are created by multiplying Gaussian noise to each bin (*v*: 0.1), by introducing insertions and deletions (*p*: 0.1) and by flipping the orientation of the peak with probability *fp*: 0.1. Individual panels (**a**–**e**) represent the different seed regions

As a proof of correctness, we constructed a simple simulation study that mimics as best as possible a real biological data set. We considered the initial 2 kb of five genes from the UCSC known genes data set, and extracted H3K4me3 data for these five regions from the ENCODE human leukaemia cell line K562 (Fig. 3). The first three of which showed bi-modal peaks, the remaining two exhibited a single peak. We ensured that the first splicing site of these five genes fell within the 2 kb region considered. We generated modified versions of the five seed regions using the following procedure (Fig. 3): A multiplicative Gaussian noise with variance *v* was applied to the read counts in each bin of a seed region. Further, each bin was removed or duplicated with probability *p* producing a shrinkage or a stretch of the peak. Bin duplication was allowed also for duplicated bins resulting in local stretching of varying length. Additionally, the orientation of the simulated peak was switched with probability *fp* in order to simulate anti-sense transcription. For each set of parameters (*v*, *p* and *fp*) we produced 99 simulated peaks starting from each seed thus obtaining a 500 peak dataset (Fig. 3).

**Fig. 4:**
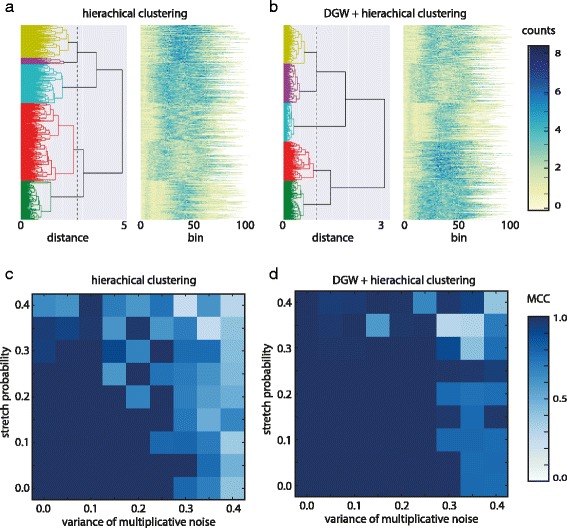
Simulation results for parameter set (*v*: 0.25, *p*: 0.25, *fp*: 0.1). **a** Left panel shows the dendrogram of clustered peaks using hierarchical clustering only. Peaks assigned to each of five different cluster are shown in *yellow*, *pink*, *blue*, *red* and *green*. X axis represents the pairwise distances **d**. Right panel shows clustered peaks. Colour coding corresponds to normalized read counts. X axis represents original (unwarped) bins from start of the peaks. **c** Matthews Correlation Coefficient for hierarchical clustering based on a set of simulations with varying parameters (*v*,*p*) and *fp* fixed to 0.1. **b** and **d** as **a** and **c** but for DGW alignment followed by hierarchical clustering

The Clustering results are shown in Fig. 4, both for a standard hierarchical clustering, as well as for DTW clustering. Figure 4 a and b show resulting dendrograms for the simulation experiment with parameters (*v*: 0.25, *p*: 0.25, *fp*: 0.1). In contrast to standard hierarchical clustering DGW identifies 5 clusters with approximately 100 members each, corresponding well to the initial five seed patterns. We reproduced the data simulation and clustering phases varying the parameter sets in order to investigate a grid of increasing modifications of peak patterns. We quantitatively assess the accuracy of the clustering using the Matthews Correlation Coefficient (MCC) with the generalization for multi-class classification problems [20,21]. The results are presented in Fig. 4 and Table 1. The MCC ranges from -1 to 1, the extreme values represent completely incorrect and completely correct classifications, respectively and 0 the result of a random classification. Standard Hierarchical clustering is able to correctly group the simulated peaks according to the pattern they are originally derived from only if the added noise and modifications are small (*v*<0.15,*p*<0.15). With DGW optimal clustering can be achieved even if the extent of local modifications to the patterns is large (Fig. 4 and Table 1).

**Table 1:** Matthews Correlation Coefficient values relative to the classifications of the synthetic peaks produced with the indicated values for *p* and *v* and *fp*=0.1

No DTW									
*p*\*v*	0.0	0.05	0.1	0.15	0.2	0.25	0.3	0.35	0.4
0.00	1.000	1.000	1.000	1.000	1.000	1.000	0.785	0.699	0.412
0.05	1.000	1.000	1.000	1.000	1.000	1.000	1.000	0.773	0.486
0.10	1.000	1.000	1.000	1.000	1.000	0.785	0.795	0.707	0.372
0.15	1.000	1.000	0.998	1.000	1.000	1.000	0.723	0.505	0.671
0.20	1.000	1.000	1.000	1.000	0.682	1.000	0.696	0.572	0.501
0.25	1.000	1.000	1.000	0.600	1.000	0.748	0.710	0.618	0.452
0.30	0.945	0.995	0.998	0.907	0.682	0.995	0.772	0.767	0.434
0.35	0.957	0.927	0.975	0.613	0.973	0.604	0.701	0.259	0.413
0.40	0.649	0.619	0.990	0.704	0.599	0.746	0.252	0.617	0.294
DTW									
*p*\*v*	0.0	0.05	0.1	0.15	0.2	0.25	0.3	0.35	0.4
0.000	1.000	1.000	1.000	1.000	1.000	1.000	0.995	0.780	0.773
0.050	1.000	1.000	1.000	1.000	1.000	1.000	0.995	0.772	0.778
0.100	1.000	1.000	1.000	1.000	1.000	1.000	0.788	0.783	0.766
0.150	1.000	1.000	1.000	1.000	1.000	0.998	0.975	0.774	0.978
0.200	1.000	1.000	1.000	1.000	1.000	0.998	0.774	0.752	0.760
0.250	1.000	1.000	1.000	1.000	1.000	0.998	0.988	0.973	0.948
0.300	1.000	0.990	0.988	1.000	1.000	0.998	0.901	0.421	0.764
0.350	1.000	0.971	1.000	0.586	0.978	0.990	0.297	0.318	0.710
0.400	1.000	0.954	0.964	0.998	0.988	0.665	0.973	0.934	0.401

### DGW automatically aligns genomic landmarks

To assess the biological significance of DGW alignment and clustering, we considered two histone marks (H3K4me3 and H3K9ac) from the ENCODE data sets. These marks were chosen as they were shown to accumulate at transcription start sites as well as first splicing sites (FSSs) [5]. Given that first exon length is highly variable, this provides a strong motivation for the local rescaling applied by DGW. For this experiment, enriched regions identified by the MACS2 peak caller were used for clustering such that no anchoring was provided. Using a bin size of 50, we restricted the analysed set of peaks to those that had a length larger than 5 and smaller than 1000 bins. Also we filtered out peaks with less than 10 counts. We used squared Euclidean distance for the local distance measure between the scaled reads and constrained the DTW with a Sakoe-Chiba Band of width 12.

**Fig. 5:**
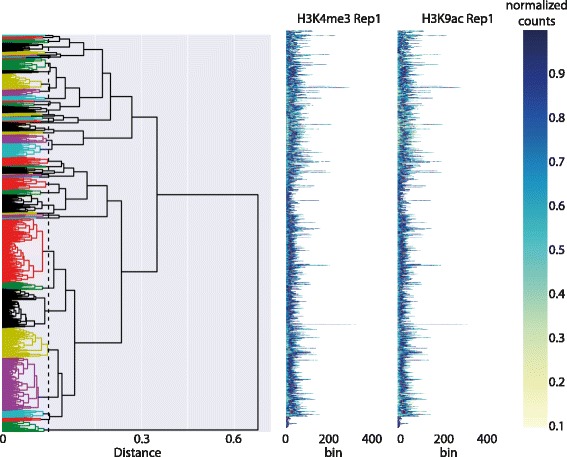
ENCODE data: DGW clustering of the H3K4me3 and H3K9ac marks in the K562 cell line. Shown are Dendrogram and heat-maps. TSS are shown as *red dots* in the heat-maps

**Fig. 6:**
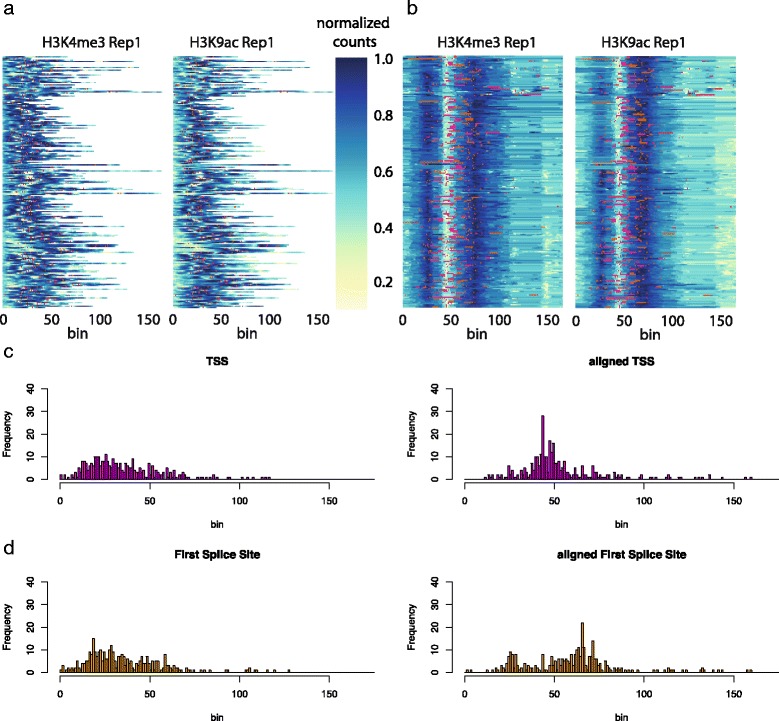
ENCODE data, a sample DGW cluster. **a** heat-maps of the raw and **b** aligned data. *Red dots* indicate transcription start sites, *orange dots* first splice sites. **c** Histograms of the positions of TSSs in raw (*left*) and aligned (*right*) data. **d** Histograms of the positions of first splice sites in raw (*left*) and aligned (*right*) data

Figure 5 shows the dendrogram and heat maps for this data. Notice the high variability in peak length, making it virtually impossible to visually distinguish any patterns. Cutting the dendrogram at an appropriate level is a difficult choice. Empirically, cutting the dendrogram near the leaves gives better visualisations, as larger clusters force the algorithm to warp together potentially very different peaks. With this in mind, we chose a cut which resulted in 45 clusters. Figure 6 a and b show the original and warped heat-maps for the two epigenomic marks within one particular cluster. TSS and First Splice Site positions are shown with red and orange dots, respectively. The heat-map of the warped data shows a well defined bimodal pattern of H3K4me3 with TSS aligning in the valley between the two sub-peaks. This is in good agreement with the known pattern of these marks around gene starts. It can be seen that these genomic landmarks or points of interest (POIs) are approximately aligned, without the usage of any prior knowledge of their position in the clustering. This is corroborated by considering the histograms of TSS and FSS positions in the raw and aligned data (Fig. 6 c and d). Computing the change in entropy between the histograms shown in Fig. 6, after rescaling the raw data to have the same length, we observe a decrease of 12.91 % for TSS and 7.72 % for FSS location distributions in the selected cluster after warping. On average, across all clusters, this effect is less pronounced, but still significant: 1.72 % decrease on average (95 % Bootstrap confidence interval 0.83 % ∼ 2.81 %) for TSS and 2.65 % (1.79 % ∼ 3.63 %) for FSS respectively, quantitatively demonstrating the ability of DGW to align these genomic landmarks.

### DGW clusters are enriched for co-factor binding sites

To probe further the biological significance of the DGW clusters, we asked whether the cluster membership could be explained in part by considering shared binding co-factors. To test this hypothesis, we considered ChIP-Seq data sets for 34 transcription factors (TFs) assayed by ENCODE in the K562 cell line (see Availability of data and materials for lists of TFs and download sources). Several TFs have been mechanistically associated with histone modifying enzymes, and indeed TF binding has recently been reported to be very strongly predictive of histone modifications [6]. We extracted peak information from these data sets, and then questioned the distribution of individual TFs binding sites across clusters. Under a reasonable null hypothesis of no relation between clustering and TF binding, one would expect the number of TF peaks falling into the genomic region corresponding to a cluster to be simply proportional to the size of the genomic region, i.e. a uniform distribution.

**Fig. 7:**
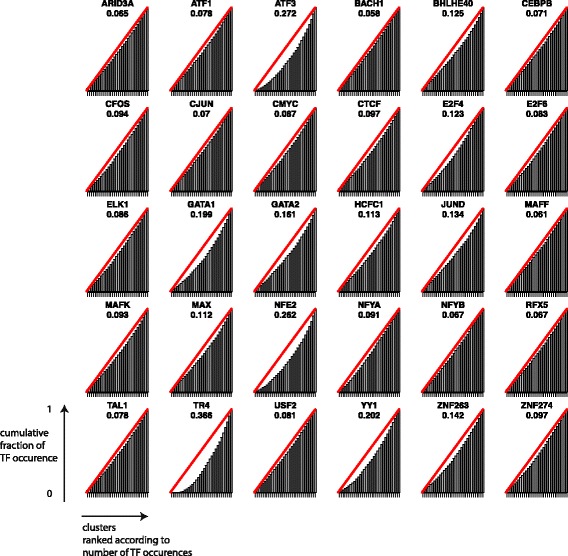
Cumulative levels of normalized overlap between each TF and the determined clusters. Each sub-plot corresponds to one TF. For each TF, clusters are ranked by their relative overlap with this TF. Each bar corresponds to the cumulative level of normalized overlap between the TF and the considered cluster plus all clusters to the left of it. The null hypothesis of uniform distribution corresponds to the *red lines*. The area between the *red line* and the cumulative plots is indicated below the TF name

Figure 7 shows normalised cumulative occurrences of TF binding sites across clusters; For each TF, clusters are ranked by their relative overlap with the given TF. Each bar corresponds to the cumulative level of normalized overlap between the TF and the considered cluster plus all clusters to the left of it. The null hypothesis of uniform distribution would correspond to the red line. On the contrary, if all binding sites for a given TF could be found in a single cluster, all bars would have length 0 except for the right most one, which would have length 1. A large area between the red line and the cumulative plot therefore indicates a strongly non-uniform distribution. Occurrence distributions for some TF, such as TR4, ATF3 or NFE2 are remarkably non-uniform and demonstrate that some clusters are highly enriched for a specific set of TFs. While these tests do not yield an immediately interpretable biological outcome, they strongly hint at a biological significance for enriched regions clustered by DGW.

## Conclusions

Data exploration and visualisation tools have played a central role in bioinformatics, and have contributed in no small part to the success of high-throughput methods in the last decade [22]. Extending these methodologies for the complex next generation sequencing data sets poses computational and methodological challenges, yet the potential for hypothesis generation is considerable. ChIP-seq data sets, in particular, yield high dimensional, structured marks associated with genomic regions. The reproducibility of the spatial structure in the ChIP-seq signal has already inspired the development of shape-based statistical tests for ChIP-seq [4]. In this paper, we addressed the natural question of whether spatial structures in ChIP-seq data can also be used to group genes with similar epigenomic marks. We have proposed a novel method, DGW, which aims to address these problems using ideas from signal processing and speech recognition. Our results show that DGW can be a practical and user friendly tool for exploratory data analysis of high throughput epigenomic data sets. DGW’s ability to recover in an unsupervised manner the observed accumulation of H3K4me3 and H3K9ac at transcription start sites and first splicing sites [5], and to associate clusters with groups of transcription factors, also demonstrates its potential as a useful tool for biological hypothesis generation. We hope that DGW may become a valuable addition to the growing toolkit for epigenome bioinformatics.
